# Characterization of N^6^-Methyladenosine in Domesticated Yak Testes Before and After Sexual Maturity

**DOI:** 10.3389/fcell.2021.755670

**Published:** 2021-11-11

**Authors:** Xingdong Wang, Jie Pei, Shaoke Guo, Mengli Cao, Pengjia Bao, Lin Xiong, Xiaoyun Wu, Min Chu, Chunnian Liang, Ping Yan, Xian Guo

**Affiliations:** Key Laboratory of Yak Breeding Engineering of Gansu Province, Lanzhou Institute of Husbandry and Pharmaceutical Sciences, Chinese Academy of Agricultural Sciences, Lanzhou, China

**Keywords:** yak, before sexual maturity, after sexual maturity, N^6^-methyladenosine, testicular tissue

## Abstract

The potential regulatory role of N^6^-methyladenosine (m^6^A), the most prominent mRNA modification in eukaryotes, has recently been identified in mammals, plants, and yeast. However, whether and how m^6^A methylation is involved in sexual maturation in mammals remains largely unexplored. In this study, testicular tissue was obtained from yaks before and after sexual maturation, and m^6^A maps were generated via preliminary experiments and methylated RNA immunoprecipitation sequencing. Only spermatogonial cells and a few primary spermatocytes were observed in the testicular tissue of yaks before sexual maturation, while spermatogenic cells at different stages of maturity could observed after sexual maturation. Experiments examining the expression of methylation-related enzymes and overall methylation levels showed that the methylation levels in yak testes increased after sexual maturation. Overall, 1,438 methylation peaks were differentially expressed before and after sexual maturation; 1,226 showed significant up-regulation and 212 showed significant down-regulation after sexual maturation. Annotation analysis showed that the differential methylation peaks were most commonly concentrated in the exon region, followed by the 3′UTR and finally the 5′UTR region. KEGG pathway analysis demonstrated that homologous recombination, the Notch signaling pathway, growth hormone synthesis, and other signaling pathways may be involved in testicular development and maturation in yaks. Levels of most m^6^A modifications were positively correlated with mRNA abundance, suggesting that m^6^A plays a regulatory role in mammalian sexual maturation. To our knowledge, this is the first report of an m^6^A transcriptional map of the yak testes, and our study lays the foundation for elucidating the function of m^6^A in the development of yak testes.

## Introduction

Yaks are a type of semi-domesticated, multifunctional cattle that became separate from other cattle about 2.2 million years ago. They are primarily found in the Qinghai-Tibet Plateau region at an altitude of 2,500–6,000 m, where there is no absolute frost-free season (Das et al., 2019a; Hui et al., 2019). The annual average temperature (−3–5°C) in plateau areas is relatively low. Animals that wish to survive in high-altitude areas need to adapt to the environments with low temperatures and oxygen levels, which is a major challenge (Hui et al., 2019). Yaks have a compact body structure, no functional sweat glands, and a relatively low skin surface area per unit body weight (0.016 m^2^/kg) (Krishnan et al., 2018). They also have a higher breathing rate, and their heart and chest cavity are more developed than those of other cattle. Furthermore, yaks have higher red blood cell counts and hemoglobin levels than other cattle, which allow better adaptation to low atmospheric oxygen levels. While these characteristics enable yaks to better adapt to freezing temperatures and hypoxic conditions, they also make them more susceptible to heat stress (Krishnan et al., 2016).

With a yak population of over 16 million (Wang et al., 2021), China houses more than 95% of all yaks in the world (Zhao et al., 2019). Yaks are multi-purpose high-altitude bovids (Das et al., 2019b) that not only provide important products such as milk, meat, fur, and fuel but are also used as local modes of transport (Wang et al., 2014; Das et al., 2019a). Yaks, which the local herdsmen call “the boat of the plateau and an all-powerful domestic animal,” thus play important social and economic roles in the plateau area, while also maintaining the pasture ecosystem and agricultural biodiversity (Wiener et al., 2003; Shah et al., 2018).

Interestingly, yaks show later sexual maturity and lower fertilization rates than other cattle (Das et al., 2019b). This low reproductive efficiency (Zhou et al., 2020), including delayed onset of puberty, seasonal reproduction, low conception rates, and long calving intervals, limits reproductive performance in yaks. Given the important role of the testes in male reproduction, research on these gonads—especially from an epigenetics perspective—could help improve the reproductive efficiency of the yak.

Epigenetic changes are potentially heritable and environmentally modifiable changes in gene expression mediated by non-DNA-encoded mechanisms (Puigoriol-Illamola et al., 2020) and mainly include DNA methylation and histone modification (Cai et al., 2021). RNA also undergoes several post-transcriptional modifications, more than 150 of which have been identified across organisms (Sun et al., 2019; Liu et al., 2020), including N^1^-methyladenosine (m^1^A), N^6^-methyladenosine (m^6^A), and 5-methylcytosine (m^5^C). These modifications are extensively found across several types of RNA, including ribosomal RNA (rRNA) and messenger RNA (mRNA) (Zheng et al., 2013b). Among these, the most common internal modification in higher eukaryotes is m^6^A, which is important for mRNA metabolism and a variety of biological processes (Liu et al., 2018).

m^6^A, discovered in the early 1970s, is a reversible and dynamic modification involving the methyltransferase complex (writer), demethylases (erasers), and binding proteins (readers) (Zhao Y. et al., 2021). Methyltransferase complexes, including methyltransferase-like 3 (*METTL3*), *METTL14*, and Wilms’ tumor 1-associated protein (*WTAP*) (Lin et al., 2017), induce methylation, which can be reversed by fat mass and obesity-associated factor (*FTO*) and AlkB homologue 5 (*ALKBH5*) (Jia et al., 2011; Zheng et al., 2013a). The function of m^6^A is dependent on its recognition by specific reader proteins bound to methylated mRNA (Wang et al., 2020). m^6^A modifications regulate almost every stage of mRNA metabolism at different levels, including RNA folding and mRNA maturation, processing, stability, output, and translation (Meyer and Jaffrey, 2014; Zhao et al., 2017). In addition, m^6^A plays a role in cell fate determination, cell cycle regulation, cell differentiation, and circadian rhythm maintenance (Wu et al., 2017).

Androgens are key for the postnatal masculinization of the fetus, especially in mammals (Rey, 2021). Hence, the process of sexual maturation is a critical period for the development of the male reproductive system. Epigenetics may influence several aspects of male reproductive tract development (Mcgowan et al., 2018), including spermatogenesis—the production of haploid sperm from diploid spermatogonial stem cells (SSCs)—which is regulated at the transcriptional, post-transcriptional, and translational levels (Lin et al., 2017). Studies in knockout mouse models have shown that *METTL3* regulates spermatogonial differentiation and meiosis initiation in mice (Xu et al., 2017). In early germ cells, the conditional knockout of *METTL3* or *METTL14* can cause the abnormal self-renewal and differentiation of SSCs (Lin and Tong, 2018). *ALKBH5* knockout mice also show impaired fertility (Wojtas et al., 2017). *FTO* mutations are positively associated with decreased semen quality, and *FTO* dysfunction may reduce male fertility (Landfors et al., 2016). Further, the deletions of *METTL3*, *METTL14*, *FTO*, *ALKBH5*, *YTHDF2*, and *YTHDC2* lead to impaired fertility or defective gametogenesis (Frye et al., 2018; Huang and Ping, 2018). Single-cell sequencing data from the studies on human testes have shown that the RNA m^6^A regulators are expressed in almost all cell types in the testes (Wang et al., 2018). Multiple studies have shown that m^6^A modification is critical for the development of the male reproductive system and especially mammalian spermatogenesis (Xu et al., 2017; Ming-Han et al., 2018). However, the regulation of m^6^A during sexual maturation in yaks, and especially its effect on spermatogenesis, remains unclear.

In recent years, new technology has allowed us to better understand RNA methylation, including m^6^A. The novel technique methylated RNA immunoprecipitation with next-generation sequencing (MeRIP-seq) was established in 2012 by two independent research groups. Subsequently, the first N^6^-methyladenosine modification map for the methylome of m^6^A RNA was generated and had a resolution of 100 nucleotides (Dominissini et al., 2012; Meyer et al., 2012). Such generation of a whole-transcriptome m^6^A methylation map via the detection of m^6^A sites at the transcriptome level could allow the further exploration of biological modifications across new areas of research and uncover the varying roles of RNA m^6^A modification.

To examine the regulatory mechanism of m^6^A modifications during the sexual maturation of yak testes, we collected healthy yak testes before and after sexual maturation and obtained the m^6^A profile of the whole transcriptome using MeRIP-seq. Differential methylation peaks were obtained by comparing maps before and after sexual maturation to elucidate the regulatory role of m^6^A methylation in yak sexual maturation.

## Materials and Methods

### Ethics Statement

All animal-related procedures conformed to the China Council on Animal Care and the Ministry of Agriculture of the People’s Republic of China guidelines. All yak-handling procedures were approved by The Animal Care and Use Committee of the Lanzhou Institute of Husbandry and Pharmaceutical Sciences Chinese Academy of Agricultural Sciences (Permit No: SYXK-2014-0002).

### Animals and Sample Collection

Testicular tissue was collected from six healthy yaks in Maqu County, Gannan Tibetan Autonomous Prefecture (33.99°N 102.07°E). Of these, three were 1.5 years old (Y group sexual maturity not reached) and three were 5 years old (M group sexual maturity reached).

#### Y Group

Testicular tissue was obtained after surgical castration. Before tissue collection, the testes were locally disinfected. After collection, the wound was sutured with a surgical needle, and penicillin and streptomycin were administered to prevent wound infection.

#### M Group

Yaks were euthanized to obtain testicular tissue. Two sets of testicles were collected from six yaks; The entire testicular tissue was cut up and placed in a cryotube that was immediately immersed in liquid nitrogen, A small portion of the testicular tissue was fixed in Bouin’s Fluid (SolarBio, Beijing, China).

Immediately after collection, samples were sent to the Lanzhou Institute of Husbandry and Pharmaceutical Sciences, Chinese Academy of Agricultural Sciences for further experiments.

### Hematoxylin–Eosin Staining

Fixed tissues were dehydrated with 75% ethanol, paraffin embedded, and then sectioned (6 mm). The sections were stained using the improved HE staining kit (SolarBio, Beijing, China) according to manufacturer’s instructions. Post-staining, the sections were sealed with neutral gum, and images were obtained on a Pannoramic 250 digital section scanner (Drnjier, Jinan, China).

### RNA Extraction and cDNA Synthesis

First, total RNA was extracted from the testicular tissue of each yak using the TRIzol reagent (Invitrogen, CA, United States). RNA concentration and purity (OD260/280 ratio) were assessed using the NanoDrop 2000 spectrophotometer (ThermoFisher Scientific, Waltham, MA, United States) and were found to be 500–5,000 ng/ml and 1.9–2.1, respectively. Furthermore, the samples were subjected to 1% agarose gel electrophoresis, and 28S and 18S rRNA bands were observed. For cDNA synthesis, each RNA sample (diluted to 500 ng/ml) was reverse transcribed using the Transcriptor First Strand cDNA Synthesis Kit (Takara Bio Inc., Dalian, China). The cDNA was subsequently stored at −80°C until further use.

### Quantitative Real-Time PCR

To study the m^6^A status in yak testicular tissue before and after sexual maturation, the levels of RNA methylation-related genes such as *METTL3*, *METTL14*, *WTAP*, *FTO*, *ALKBH5*, *YTHDF1/2/3*, *YTHDC1/2*, *RBM15*, *VIRMA*, and *ZC3H13* were detected using qRT-PCR. The National Center for Biotechnology Information website was used for primer designing (Supplementary Table S1). RT-PCR was performed using a CFX Link Real-Time PCR Detection System. The reaction volume was 20 μl, including 10 µl of 2× PrecisionPLUS Master Mix (Primerdesign), 1 µl of diluted cDNA (25 ng), 1 µl (300 nmol) of gene-specific forward and reverse primers each, and 7 µl of RNase/DNase free water. The standard PCR reaction conditions for all transcripts were as follows: 95°C (3 min), followed by 39 cycles of 95°C (10 s) and 55°C (30 s). *GAPDH* was used as the reference gene, and relative gene expression was examined using the 2-^ΔΔCT^ method (Schmittgen, 2001). Each reaction was repeated in triplicate to obtain Ct values. Analysis of variance (ANOVA) was used to analyze differences in the expression of methylation-related enzymes.

### Evaluation of m^6^A Content

The total mRNA m^6^A levels in the yak testes were estimated using the EpiQuik RNA Methylation Quantitative Kit (Epigentek, P-9005, NY, United States). An m^6^A standard curve (0.01–0.5 ng/μl) was constructed based on the manufacturer’s instructions for the kit. Absorbance was measured using a microplate (Thermo Scientific., Shanghai, China) reader at 450 nm.

### MeRIP-Seq and mRNA Sequencing

First, the quality and quantity of total RNA were estimated using Bioanalyzer 2,100 (Agilent, CA, United Stets) and NanoDrop 2000 (Thermo Scientific), both with an RIN number > 7.0. More than 100 μg of total RNA was used for mRNA isolation via an mRNA Purification Kit (Ambion Dynabeads mRNA Purification Kit). Subsequently, poly (A) mRNA fractions were created using divalent cations and a thermocycler (RNA solution kept at 94°C for exactly 5), incubated with m^6^A-Dynabeads (Anti-m^6^A, Synaptic Systems, Cat. No 202003) in an m^6^A-binding buffer (50 mM Tris-HCl pH 7.4, 150 mM NaCl_2_, 1% NP-40, 2 mM EDTA), and allowed to bind to the beads. Following this, the m^6^A-Dynabeads were washed and m^6^A-positive RNA was eluted. The RIP was extracted and cleaned, and finally, m^6^A “enriched RNA” was collected. We used 100 ng of RNA (100 ng of input and 100 ng of post m^6^A-IP positive fraction) for library construction with the Illumina TrueSeq Stranded mRNA platform. Finally, paired-end sequencing was performed on an Illumina HiSeq X10 system at OE Biotech Co., Ltd. (Shanghai, China) using manufacturer’s instructions. The data was submitted to the GENE EXPRESSION OMNIBUS (GEO) database (accession number GSE181266).

### Analysis of Sequencing Data

Raw reads obtained from RNA sequences were subjected to statistical analysis and quality control (Supplementary Table S2). Raw data (raw reads) in the fastq format were processed using the Trimmomatic (Bolger et al., 2014) software, and reads with adapter sequences or poly-N sequences as well as low-quality reads were removed to obtain clean data. Following this, 250,000 paired reads were randomly extracted from the clean data, and blastn was used with the NT database (ftp://fp.ncbi.nih.gov/blast/db) to examine sequence alignment with the reads. The most high-quality results with an e value < 1e-10 and coverage > 80% were selected. Meanwhile, the SortMeRNA (Evguenia et al., 2012) software was used for removing ribosomal RNA reads. The remaining clean reads were mapped to the reference genome, LU_Bosgru_v3.0 (ftp://ftp.ensembl.org/pub/release-99/fasta/bos_grunniens/dna/Bos_grunniens.LU_Bosgru_v3.0.dna_sm.toplevel. Fa. gz) for sequence alignment using HISAT2 (Kim et al., 2015) to obtain position information on the reference genome as well as specific sequence characteristic information from the sequenced samples. Default parameters were used, and unique reads showing high mapping quality were retained. The Guitar (Cui et al., 2016) R package and deeptools (Fidel et al., 2014) software were used to evaluate m^6^A-seq data quality in order to assess the quality of MeRIP-seq data.

m^6^A-enriched peaks were detected in each ample using the MeTDiff peak calling software (screening criteria: p <= 0.05; fc >= 1.5) and the options FRAGMENT_LENGTH = 200, PEAK_CUTOFF_PVALUE = 0.01, and PEAK_CUTOFF_FDR = 0.05) (Cui et al., 2018). The number, width, and distribution of peaks were statistically analyzed, and the corresponding input sample was used as the control. The identified peaks were annotated based on their intersection with the gene architecture using ChIPseeker (Yu et al., 2015). Finally, differential analysis of m^6^A-seq data was performed by comparing data from yaks who had yet to reach sexual maturation with that from yaks who had already reached sexual maturation. This was done using MeTDiff (screening criteria: diff. p <= 0.05; diff. fc >= 1.5) with the following parameters: FRAGMENT_LENGTH = 200, PEAK_CUTOFF_PVALUE = 0.01, DIFF_PEAK_CUTOFF_FDR = 0.05, and PEAK_CUTOFF_FDR = 0.05). The differential peaks were again annotated using ChIPseeker.

Gene ontology (GO) enrichment and Kyoto Encyclopedia of Genes and Genomes (KEGG) pathway enrichment analyses of the identified peaks and differential peaks were performed using R based on hypergeometric distribution. Sequence motifs were identified using MEME (Bailey et al., 2009) and DREME (Schulz et al., 2012) and annotated using Tomtom software.

### Statistical Analysis

All statistical analyses were performed using SPSS version 21.0 (Qiu et al., 2007). One-way ANOVA was used to analyze between-group differences. *p* < 0.05 or *p* < 0.01 was considered statistically significant (Lan et al., 2013).

## Results

### HE Staining of Testicular Tissue

**FIGURE 1:**
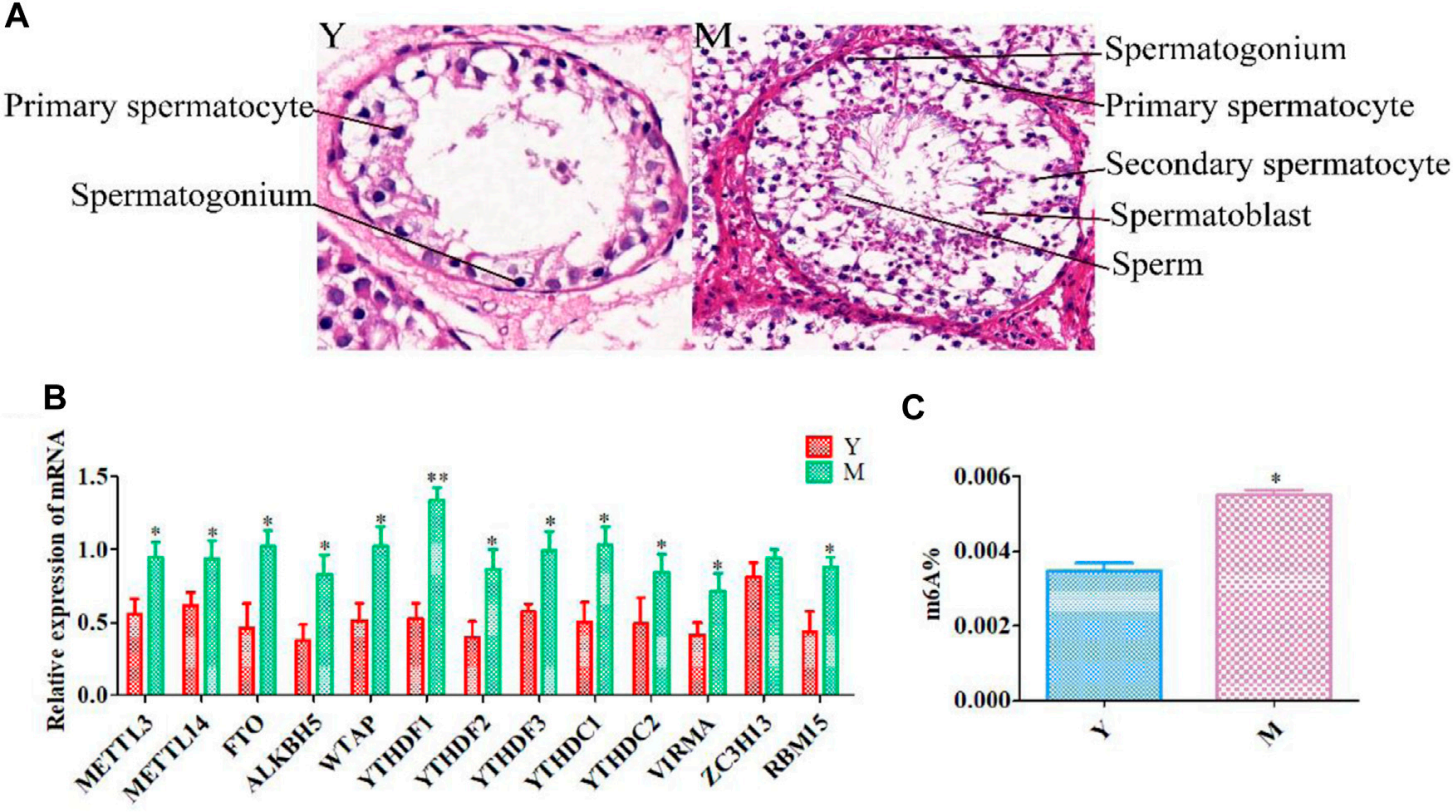
Histological and qRT-PCR analysis. **(A)** HE staining of yak testes before **(Y)** and after **(M)** sexual maturation. **(B)** Quantitative expression of methylation-related genes. **(C)** Overall RNA methylation levels in testicular tissue. **p < 0.01, *p < 0.05.

Testicular tissue sections (Figure 1A) of yaks at the pre- (Y) and post (M)-sexual maturation stage were examined. Only spermatogonia and a few primary spermatocytes could be observed in yak testes before sexual maturation. However, after sexual maturation, spermatogenic cells at all stages of development were found to be distributed in the yak testes. Moreover, sperm cells were detected in the middle of the seminiferous tubules.

### Quantitative RT-PCR Findings and Global Methylation Levels

After sexual maturation, demethylases (*FTO* and *ALKBH5*), methyltransferases (*METTL3*, *METTL14*, *WTAP*, *VIRMA*, and *RBM15*), and m^6^A-binding proteins (*YTHDC1*, *YTHDC2*, *YTHDF2*, and *YTHDF3*) were significantly up-regulated. Moreover, while *YTHDF1* showed very significant up-regulation after sexual maturation, *ZC3H13* expression did not change significantly (Figure 1B). Such changes in the expression of methylation-related enzymes could result in dynamic variations in m^6^A methylation in testicular tissue after sexual maturation in yaks. Therefore, we compared the methylation status of yak testicular tissue before and after sexual maturation and observed a significant increase in m^6^A levels after sexual maturation (Figure 1C).

### Sequencing Quality Control and Reference Genome Alignment

The available data volume of each sample ranged from 6.07G to 6.83G, the Q30 base distribution ranged from 92.63 to 93.68%, and the average GC content was 48.61%. The genome alignment rate of each sample with respect to the reference genome was 88.62–94.21% (Supplementary Table S3). A high multiple comparison rate was observed in the comparison analysis, and multiple alignment reads were therefore excluded. Only single reads were retained for subsequent analysis. Due to some differences in the matching rate of the samples, the results of matching were divided into three parts: unaligned reads, uniquely mapped reads, and multiple mapped reads (Supplementary Figure S1A).

### Methylation Peak Detection and Annotation

We obtained an m6A map of the complete yak transcriptome using high-throughput sequencing and analyzed the detected peaks (Supplementary Table S4; Supplementary Figure S1B). In group Y (1.5-year-old yaks), the number of peaks was 10,042, and genome percentage was 1.54%. In group M (5-year-old yaks), the number of peaks was 11,763, and the genome percentage was 1.87%. Due to the differences in the predicted methylation sites across different samples, the predicted methylation sites were divided into five categories based on reliability: none, low, moderate, high, and very high (Supplementary Figure S1C).

**FIGURE 2:**
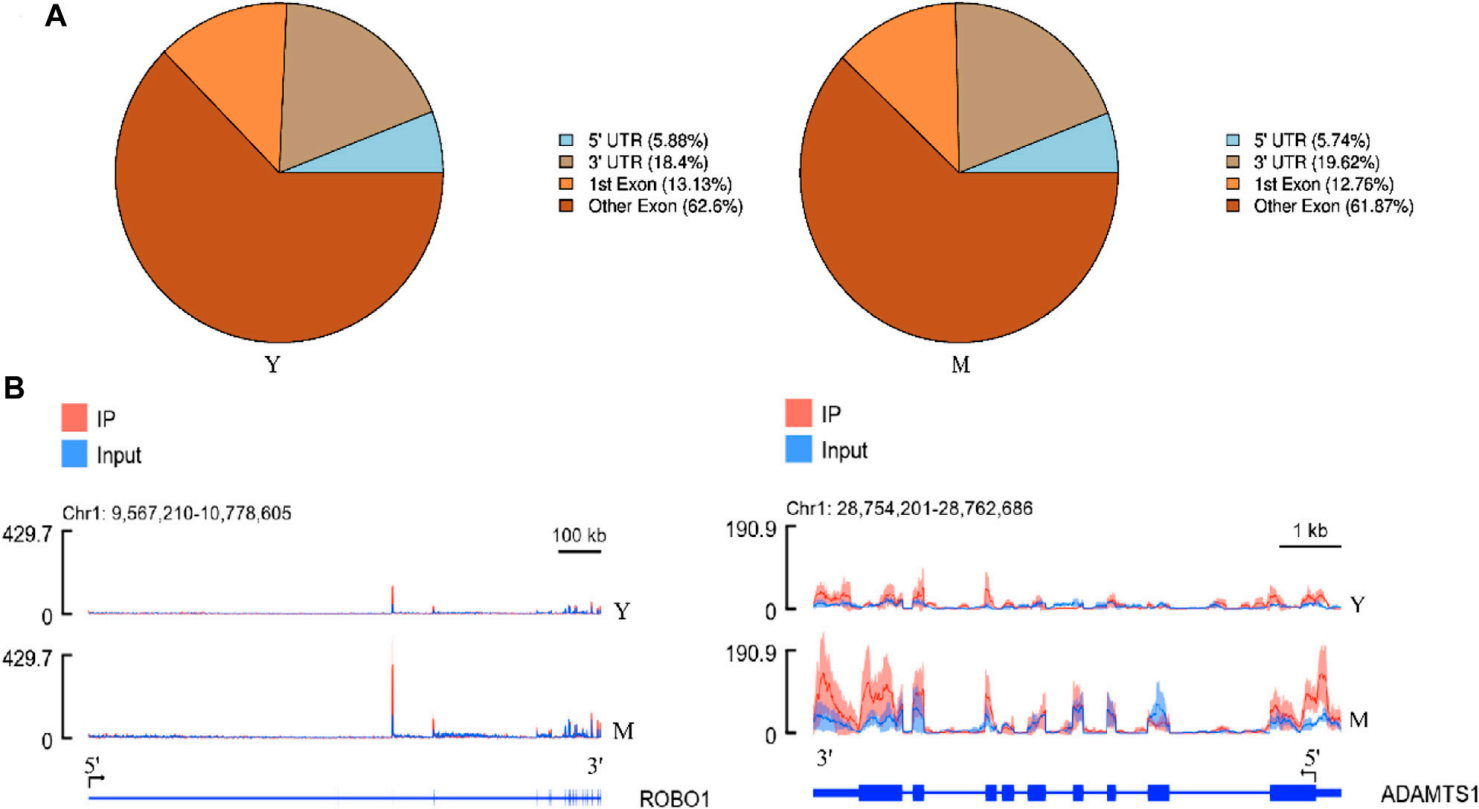
Topological distribution of m6A peaks. **(A)**, Pie chart showing the peak in gene functional element region annotation. **(B)**, IGV plot shows directly the peaks in the genes of ROBO1 and ADAMTS1. The peak of ROBO1 was located at the CDS and 3′ UTR regions, and the peak of ADAMTS1 was located at the 3′ UTR, the CDS, and the 5′ UTR regions.

**FIGURE 3:**
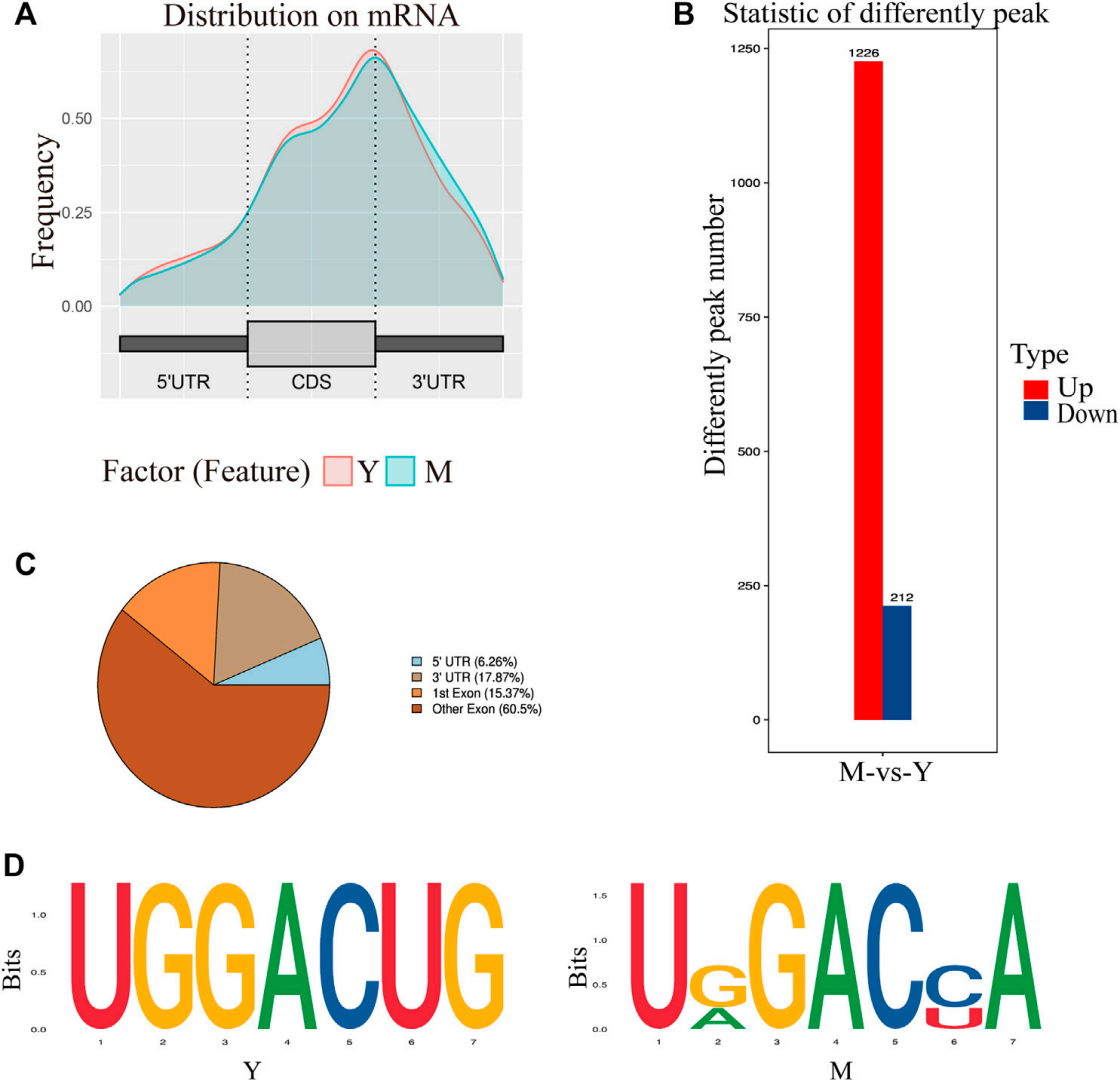
Characteristics of the m^6^A peak. **(A)** Distribution of m^6^A peak on mRNA. The peaks were mostly distributed on the exon region and there was a distinct enrichment peak at the 3′ terminate (near the stop codon). Y: Before sexual maturity, M: After sexual maturity. **(B)** Statistical analysis of differential peaks. **(C)** Distribution of differential methylation peaks on mRNA. **(D)** Top motifs with m^6^A peaks in the M and Y groups. This is based on the respective high-trust peak analysis in each group.

Annotation results from ChIPseeker were counted. We observed 1–6 m6A peaks on each gene, and 85% of genes had only one peak. (Supplementary Table S5). The gene *FREM2* located on chromosome 15 had the most methylation peaks (6 m^6^A peaks). Statistical analyses of peak width showed that peaks of 0–1,000 bp were the most common, and those sized more than 100,000 bp were the least common (Table 1). The majority of peaks were concentrated in the exon region, followed by the 3′UTR and 5′UTR region (Figure 2A). We have chosen two genes to show the m6A methylation pattern. The peak of ROBO1 was located at the CDS and 3′ UTR regions, and the peak of ADAMTS1 was located at the 3′ UTR, the CDS, and the 5′ UTR regions (Figure 2B). A significant enrichment was observed in peaks at the 3′UTR (near the stop codon) (Figure 3A).

**TABLE 1:** Statistical analyses of peak widths.

Peaks width (bp)	M (number)	Y (number)
1–1,000	5,584	6,540
1,000–5,000	2,606	3,045
5,000–10,000	877	1,043
10,000–100,000	936	1,086
>100,000	37	47

Differential peak analysis revealed 1,438 differential methylation peaks between the M and Y groups. The total length of these peaks was 5,352,512 bp and their average length was 3,722.19 bp, accounting for 0.19% of the genome. The number of significantly up-regulated and down-regulated methylation peaks in Y-VS-M was 1,226 and 212, respectively (Figure 3B). Annotation analysis revealed that the differential methylation peaks were also mainly concentrated in the exon region, followed by the 3′UTR and finally the 5′UTR region (Figure 3C).

Next, we determined whether the identified m^6^A peaks shared any common motifs. We found that m^6^A modifications were typically present in 5-RRACH-3 (R = A or G; H = A, C, or U) sequences (Figure 3D), as reported previously (Yi et al., 2021).

### GO and KEGG Pathway Analysis

**FIGURE 4:**
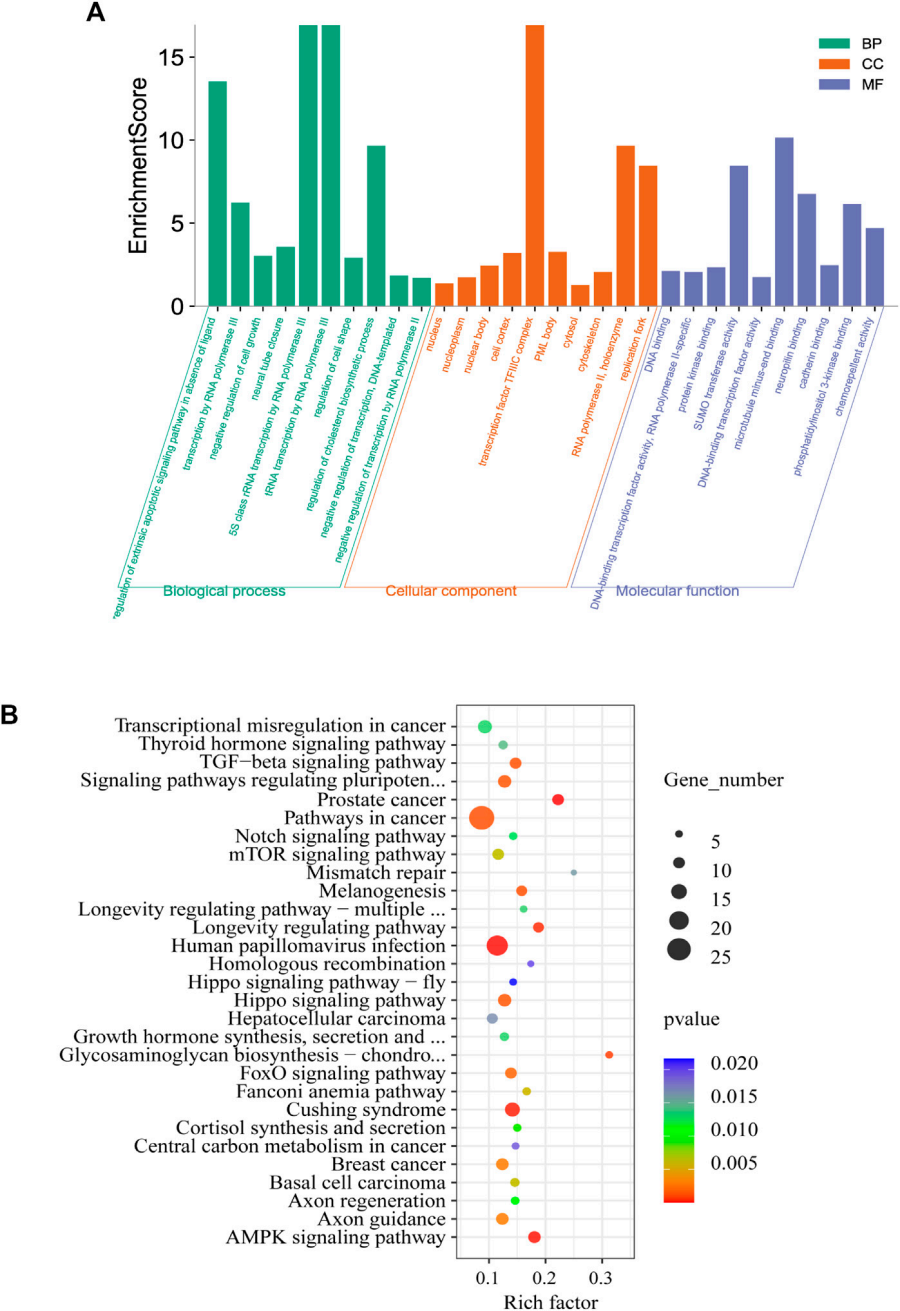
Enrichment analysis. **(A,B)**, GO **(A)** and KEGG **(B)** analysis of genes associated with differential methylation peaks before and after sexual maturation.

To elucidate the function of m^6^A RNA methylation in testicular sexual maturation, we performed functional enrichment analyses of genes with significantly altered m^6^A methylation levels based on the GO and KEGG databases. GO analysis showed enrichment for m^6^A methylation in the following processes across the three biological modules of GO: 1) Molecular function: protein kinase binding, phosphatidylinositol 3-kinase binding, and cadherin binding; 2) Cellular component: nucleoplasm, cytoskeleton, and cell cortex; 3) Biological process: transcription by RNA polymerase III, regulation of cholesterol biosynthesis, and neural tube closure (Figure 4A). KEGG analysis showed that genes with significant changes in m^6^A methylation levels were mainly enriched in processes such as homologous recombination, the Notch signaling pathway, and Growth hormone synthesis, secretion, and action (Figure 4B).

**FIGURE 5:**
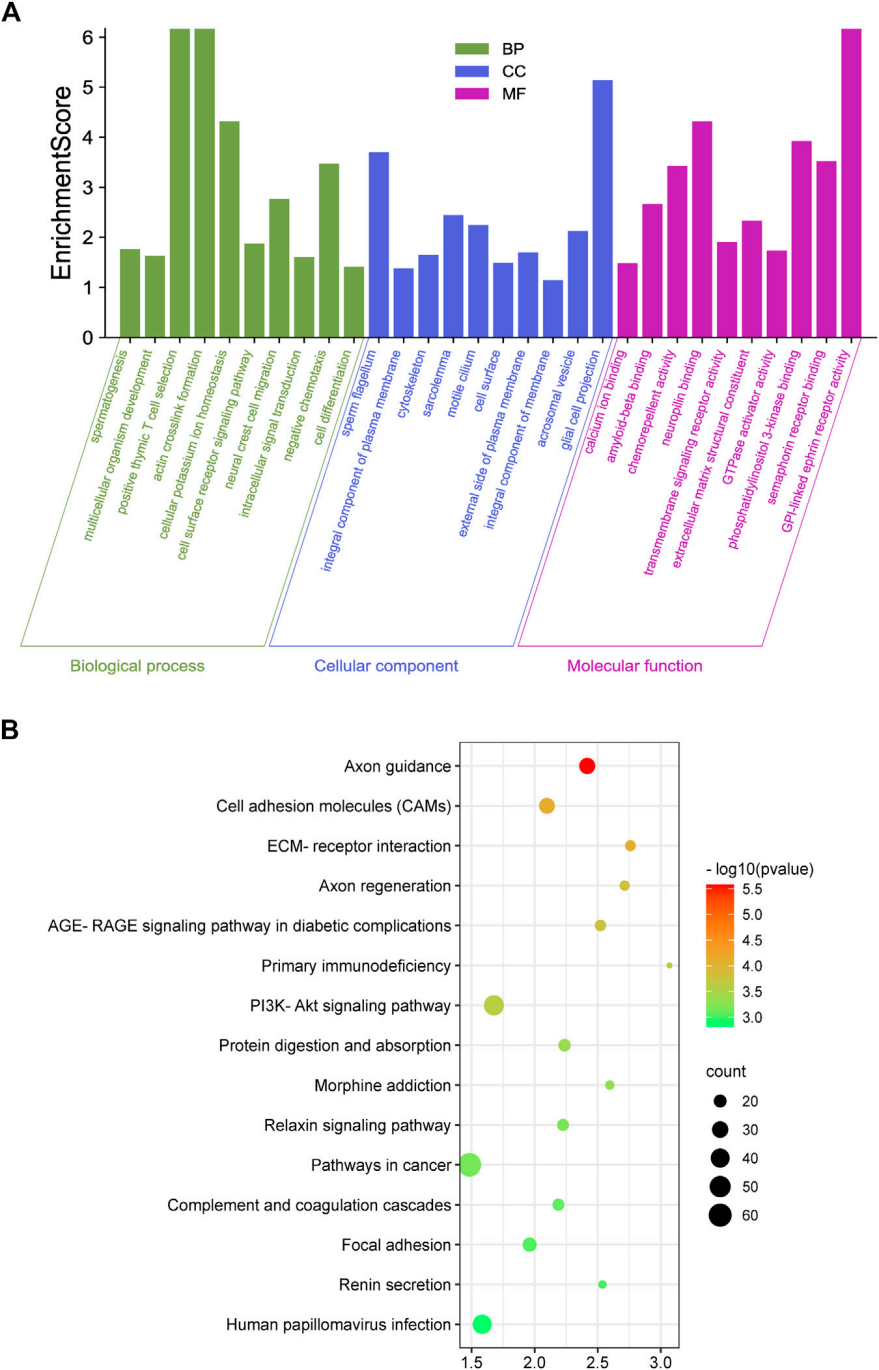
Enrichment analysis. **(A,B)**, GO **(A)** and KEGG **(B)** enrichment analysis of genes differentially expressed before and after sexual maturation.

We examined mRNA-level changes in the testicular tissue of yaks before and after sexual maturation based on RNA-seq data (Supplementary Table S6). Compared with the Y group, 895 and 760 genes were up- and down-regulated, respectively, in the M group (*p* < 0.5, log2FC > 1), indicating that mRNA expression levels changed during sexual maturation. Further analysis of these differentially expressed genes using GO analysis showed enrichment in the following processes: 1) Molecular function: transmembrane signaling receptor activity, semaphorin receptor binding, and phosphatidylinositol 3-kinase binding; 2) Cellular component: sperm flagellum, motile cilium, and cytoskeleton; 3) Biological process: spermatogenesis, cell differentiation, and actin crosslink formation (Figure 5A). KEGG analysis revealed that the differentially expressed genes had significant enrichment in pathways such as Axon guidance, the PI3K-Akt signaling pathway, the Relaxin signaling pathway, and Focal adhesion (Figure 5B).

By comparing the GO and KEGG analysis results of differentially expressed genes and differential methylation of corresponding genes, we found enrichment in the GO terms for phosphatidylinositol 3-kinase binding and other kinase binding, cytoskeleton, and spermatogenesis. KEGG analysis showed that all of the genes were enriched in spermatogenesis-related signaling pathways. These results indicated that the sexual maturity-related changes in gene expression and methylation levels in yaks were associated with spermatogenesis.

### Combined Comparison of m^6^A Methylation and Gene Expression Before and After Sexual Maturation

**FIGURE 6:**
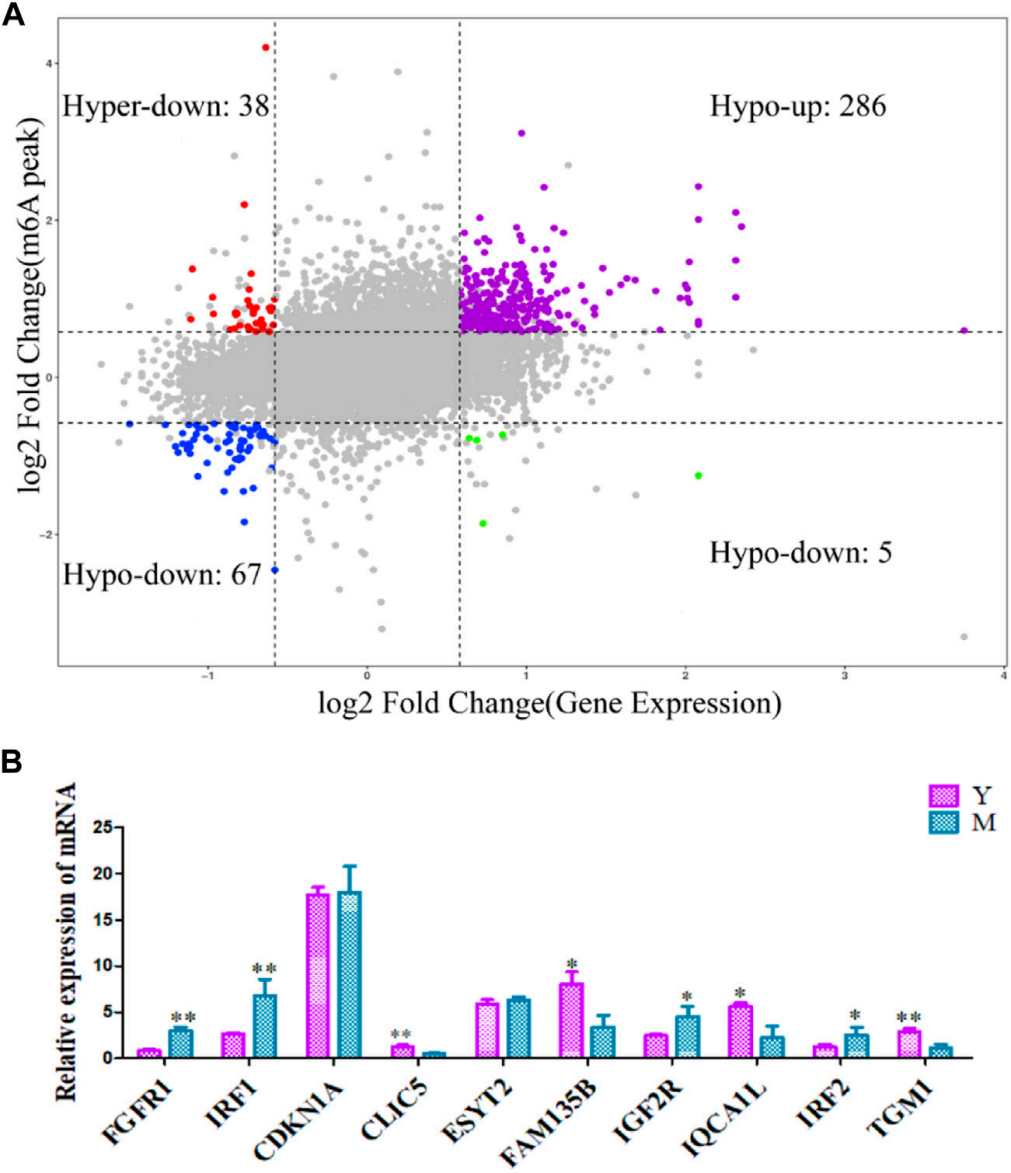
Differentially methylated and expressed genes examined using conjoint analysis and verified using qRT-PCR. **(A)** Combined four-quadrant scatter plot of genes showing differential methylation and differential expression (gray dots, genes with no significant differences; colored dots, genes with significant differences). **(B)** The differentially expressed genes were verified using qRT-PCR. Y: before sexual maturation. M: after sexual maturation. **p < 0.01, *p < 0.05.

To further elucidate the relationship between m^6^A methylation and RNA expression levels, we used peaks with a Log2 Fold Change >0.5, *p* < 0.01 and mRNAs showing a log2 foldchange >0.5, *p* < 0.01. The difference in the m^6^A methylation level was less than 0.05. Both m6A peaks and mRNA levels were significantly different for 396 mRNAs. Of these, 286 showed an up-regulation of m^6^A peaks and mRNA expression, 38 showed an up-regulation of m^6^A peaks and down-regulation of mRNA expression, 5 showed a down-regulation of m^6^A peaks and up-regulation of mRNA expression, and 67 showed a down-regulation of both m^6^A peaks and mRNA expression (Supplementary Table S7). The association of m^6^A methylation with mRNA expression is depicted in Figure 6A.

### Verification of m^6^A-Modified Differentially Expressed Genes

qRT-PCR was used to verify the levels of differentially methylated genes in yak testes before and after sexual maturation. The observed expression patterns corroborated the RNA-seq data (Figure 6B), confirming the validity of our transcriptome analysis.

## Discussion

Sexual maturation is a continuous process that allows males to produce mature sperm and maintain fertility. Spermatogenesis, a complex developmental process, is meticulously controlled by complex regulatory processes at several levels (Lin and Tong, 2018). m^6^A RNA modification is critical for the development of the male reproductive system (Zhao T. et al., 2021). The development of testicular tissue is a hot-spot in reproductive biology research. In our study, HE staining was performed on testicular tissue sections. This staining revealed significant differences in testicular tissue before and after sexual maturity. This was consistent with experimental findings from Qiu et al., who observed no sperm in smears prepared using testicular tissue from 1.5-year-old Datong yak testicles but observed normal sperm in those prepared using testicles obtained from yaks aged 2 years or older (Qiu et al., 2010). These results indicated that the stages at which we obtained yak testicular tissue in the present study were suitable for the purpose of our research.

qRT-PCR confirmed that methylation-related enzymes in the yak testes showed different levels before and after sexual maturation. *ALKBH5*, *YTHDC2*, *FTO*, *METTL3*, and *METTL14* showed significantly higher levels after sexual maturation. Murine studies have shown that *ALKBH5* knockout results in impaired spermatogenesis and male sterility (Zheng et al., 2013a). In *YTHDC2* knockout mice, germ cells do not mature beyond the zygotic period (Hsu et al., 2017). *METTL3* and *METTL14* double-knockout down-regulates the translation of the key spermatogenesis-related m^6^A modification transcripts, leading to anomalies in spermatogenesis during anaphase (Lin et al., 2017). *FTO* mutations are positively associated with decreased semen quality and may reduce male fertility (Landfors et al., 2016). Therefore, it appears thar m^6^A methylation-related enzymes are vital for maintaining the normal function of the male reproductive system. We also observed that the overall methylation levels in yak testicular tissue increased after sexual maturation. This was in line with results from Sun et al.’s (2020) study , in which methylation levels were observed to increase with age in mice, peaking in adult males.

In mammals, m^6^A enrichment is observed near the stop codon and the 3′UTR. Ke et al. (2015) found that most m6A residues are located in the last exon, allowing for 3′UTR regulation. In contrast, in amphibians such as *Xenopus laevis,* m^6^A enrichment is observed near the start and stop codons (Sai et al., 2020). By analyzing the distribution of methylation peaks in the yak testes, we found m^6^A peaks to be primarily enrichmented around the 3′UTR near the stop codon, consistent with the results of human and mouse studies (Dominissini et al., 2012; Meyer et al., 2012; Schwartz et al., 2014). Different genes of last Exon are different and the statistical difficulty is high, so the experimental statistics are only divided into two parts, namely the first exon and other exons. Hence, the overall distribution of m^6^A sites appears to be comparable across mammals. m^6^A has been found to contain a conserved RRACH motif (R = a purine, A = m^6^A, and H = non-guanine base) (Dominissini et al., 2012; Meyer et al., 2012). By analyzing the enrichment motifs around the m^6^A peak, we also found a large number of RRACH motifs, consistent with the results observed in plants, mammals, yeast, and amphibians (Sai et al., 2020). This strongly suggests that RNA adenosine methylation is conserved across mammals.

m^6^A modifications are known to generate mRNA instability (Geula et al., 2015). To elucidate the role of m^6^A methylation in sexual maturation, we probed the different expression peaks across differentially expressed mRNAs. A total of 396 mRNAs showed differential expression peaks, 353 of which showed patterns consistent with the trends of peak expression, accounting for 89% of all the identified mRNAs. m^6^A peaks showed a positive correlation with the expression of related genes.

Subsequently, we delineated the role of m^6^A modifications in sexual maturation. GO annotation showed that most mRNAs containing m^6^A modifications were enriched in different domains across the molecular function, cellular component, and biological process modules, and their functions were mainly concentrated in aspects such as binding and catalytic activity. KEGG analysis showed that differential m^6^A modification was primarily associated with signal transduction and hormone synthesis. Androgenic action is key for the postnatal masculinization of the fetus, especially in humans and other mammals, and the preadolescent stage is characterized by a marked lack of gonadal steroid secretion. During pubertal development, the testes resume androgen production, and the effects of androgens become apparent during the development of male secondary sexual characteristics (Rey, 2021). Hence, m^6^A modifications occur not only in insects and during virus infection (Zhang et al., 2019), but also in the testes during yak sexual maturation.

We observed that m^6^A methylation levels of genes in testicular tissue changed significantly after sexual maturation in the yak testes. UBE3A is a ubiquitin-linked protease E3A. Ubiquitin is known to regulate many important biochemical processes in the testes, including DNA repair, structural control of meiotic chromatin (Grootegoed et al., 1998), and histone protamine transformation during spermatogenesis (Nickel et al., 1989). Previous studies have suggested that the candidate genes for male infertility are involved in ubiquitination. Wong et al. (Wong et al., 2002) confirmed the specific interaction between VCY2 and UBE3A through yeast two-hybridization and *in vitro* co-immunoprecipitation and also confirmed that both the *VCY2* and *UBE3A* genes are located in the germ cell compartment. *VCY2*, present in the AZFC region of the YQ chromosome, is often absent in infertile men with severe oligozoospermia or azoospermia. These data suggest that UBE3A ubiquitination may be necessary for the function of VCY2 and that UBE3A and VCY2 act synergically to regulate spermatogenesis.

The presence of m^6^A is essential for normal animal development. KEGG analysis revealed that genes showing differential m^6^A mRNA methylation play a role in several biological pathways, many of which are involved in male reproduction. The evolutionarily conserved Notch pathway has been found to regulate cell fate determination in some tissues (Borggrefe and Oswald, 2009). The expression of Notch pathway components has been reported in neonatal and adult mammal testes (Dirami et al., 2001; Hayashi et al., 2001). In addition, this pathway is involved in germ cell development in *Caenorhabditis elegans* (Kimble and Crittenden, 2007) and *Drosophila melanogaster* (Song et al., 2007). Notch pathway activity in Sertoli cells is known to be critical for determining germ cell fate during the early development of testes. It is also crucial for regulating the differentiation of spermatogonia in adult testes (Garcia et al., 2017), and its interruption leads to increased apoptosis and spermatogenesis defects (Murta et al., 2014). Abnormal Notch activity is also associated with male infertility in rodents and humans (Hayashi et al., 2001; Hayashi et al., 2004). Findings from Murta et al. (Murta et al., 2013) strongly suggest that Notch signaling helps maintain the spermatogonial cell bank, initiation of spermatogenesis, regulation of spermatogenesis speed, maintenance and differentiation of germ cells, and function of mesenchymal cells.

Although methylation-related enzymes are known to play an important role in spermatogenesis and an m^6^A transcriptional map of sexual maturation in yaks has been developed, how changes in methylation levels affect sexual maturation in mammals remains unclear. The methylation levels in yak testes before and after sexual maturity were measured at the overall tissue level in the present study. However, testicular tissue contains different cell types at different stages, and it is currently challenging to specifically identify which cells show changes in methylation levels. Therefore, understanding the regulation of methylation level changes during the process of sexual maturation is difficult. Currently, there are technical and experience-related limitations in isolating and culturing the different types of cells from yak testes, which creates further problems in elucidating how methylation precisely controls sexual maturation in these animals. In the future, we aim to isolate different cell types from yak testicular tissue using enzymatic hydrolysis and thereby establish an *in vitro* culture system. By detecting methylation levels in these cell types and perturbing the key methylation regulators in the process of sexual maturation, we hope to further delineate the effect of methylation on sexual maturity.

## Conclusion

In this study, the methylation levels in yak testicular tissue were compared before and after sexual maturation and gene enrichment analyses were conducted. We found that methylation levels increase in yak testes after sexual maturation and that several genes showing differential m^6^A expression are related to male reproduction. To our knowledge, this is the first report of an m^6^A transcriptional map of the yak testes, and our study lays the foundation for elucidating the function of m^6^A in the development of yak testes. Although the mechanism underlying post-transcriptional gene regulation and its biological significance in the process of sexual maturation in yaks are not currently clear, functional studies on methylation-related genes and the signaling pathways they are involved in should be carried out to confirm their significance in yak sexual maturation in the future.

## Data Availability

The datasets presented in this study can be found in online repositories. The names of the repository/repositories and accession number(s) can be found in the article/Supplementary Material.
