# Supplementary material for: Characterization of N6-Methyladenosine in Domesticated Yak Testes Before and After Sexual Maturity
Source: Front Cell Dev Biol. 2021 Nov 11;9:755670. doi: 10.3389/fcell.2021.755670 (PMC8632223; doi:10.3389/fcell.2021.755670)
Supplement: Supplementary file 3 [file Table1.DOCX]

Supplementary Table S1. Primer sequence.

| **Gene** | **Primer sequences (5' to 3')** | **TM/ ^o^C** |
| --- | --- | --- |
| METTL3 | F: TAGCCAAGGAGCCAACCAAG  R: TCTTGAACTTGAGCCCGACC | 57 |
| METTL14 | F: TCTGGGGAAGGATTGGACCT  R: CCCGTCTGTGCTACGCTTTA | 57 |
| WTAP | F: GTCTGGATTTCACAGGGAGGG  R: CAGGTTTCTCCTCTTGCGGG | 59 |
| FTO | F: GCACAAGCACGGCTGTTTAT  R: TTGACAGGCGGCAGCTATTT | 55 |
| ALKBH5 | F: ACGAGATTAGATGCACCCCG  R: CTGTTGTTTCCCGACAGACG | 57 |
| YTHDF1 | F: GTCACCACCTCTGCAGTCAA  R: TGTTTAATAGGTGGCGGGGG | 57 |
| YTHDF2 | F: CTGCGAAACAGCAACCCAAG  R: TGCCACAGGACCCTTGTTATC | 57 |
| YTHDF3 | F: CAGAGGAAACAGGCGAAGAA  R: CTGCTTCCCCAAGCGAATATG | 57 |
| YTHDC1 | F: GAAGCTCTGCATCGGAGTCA  R: CTTTTCGGACAGCACGAACG | 57 |
| YTHDC2 | F: AGTGGAAGTGATCTCAGTGCAG  R: GTGCATTTTCAGCAAACCGC | 56 |
| RBM15 | F: GAGTTTGACCGGTTTGGCAC  R: TGAGTTAACGGCAGAGGCTG | 57 |
| ZC3H13 | F: CCCCAGGGATTCTCGGTCTA  R: CTACTTCTGCCCATCCGGTC | 59.5 |
| VIRMA | F: ACTACTGCCTTGCGTGTTCT  R: AGCGAGCCATCGAAATGGTA | 55.4 |
| FGFR1 | F: GCCTGTACACAGGATGGTCC  R: TGTCTCGAGCAAGACCGAAG | 59 |
| IRF1 | F: ACACAGGCCGATACAAAGCA  R: TCGGCTGGACTTGGACTTTC | 57 |
| IRF2 | F: ACCTGACCCAAAAACGTGGA  R: GTCAGGACCGCATACTCAGG | 57 |
| IGF2R | F: CCGTGTGTGCCGTGGATAAG  R: GGAGTCAAAGACCGTGCAGT | 59 |
| TGM1 | F: TTCACGCTCAGACCAGAACC  R: ATTTCCGATGCACAGCTCCA | 57 |
| IQCA1L | F: TCTCAGAGGGGAGCTTTCCA | 59 |
|  | R: GAATCTTCCGGTCTCGCTCC |  |
| CLIC5 | F: TGCGAAACATCGGGAATCCA | 57 |
|  | R: AGCCCTTTTCATCATCCCCG |  |
| FAM135B | F: CTGTCGGTGATCTCGGTGAC | 59 |
|  | R: CCAGGGAAAGGCTAGACTGC |  |
| CDKN1A | F: GCGGTGGAACTTCGACTTTG | 57 |
|  | R: ATCTGTCATGCTGGTCTGCC |  |
| ESYT2 | F: GTAGAGCTGGTGTGCAGAGTA | 57 |
|  | R: CCGTGATTCGATTGGGAAGC |  |
| GAPDH | F: TCACCAGGGCTGCTTTTA | 60 |
|  | R: CTGTGCCGTTGAACTTGC |  |
