# Supplementary material for: Characterization of N6-Methyladenosine in Domesticated Yak Testes Before and After Sexual Maturity
Source: Front Cell Dev Biol. 2021 Nov 11;9:755670. doi: 10.3389/fcell.2021.755670 (PMC8632223; doi:10.3389/fcell.2021.755670)
Supplement: Supplementary file 8 [file Image1.pdf]

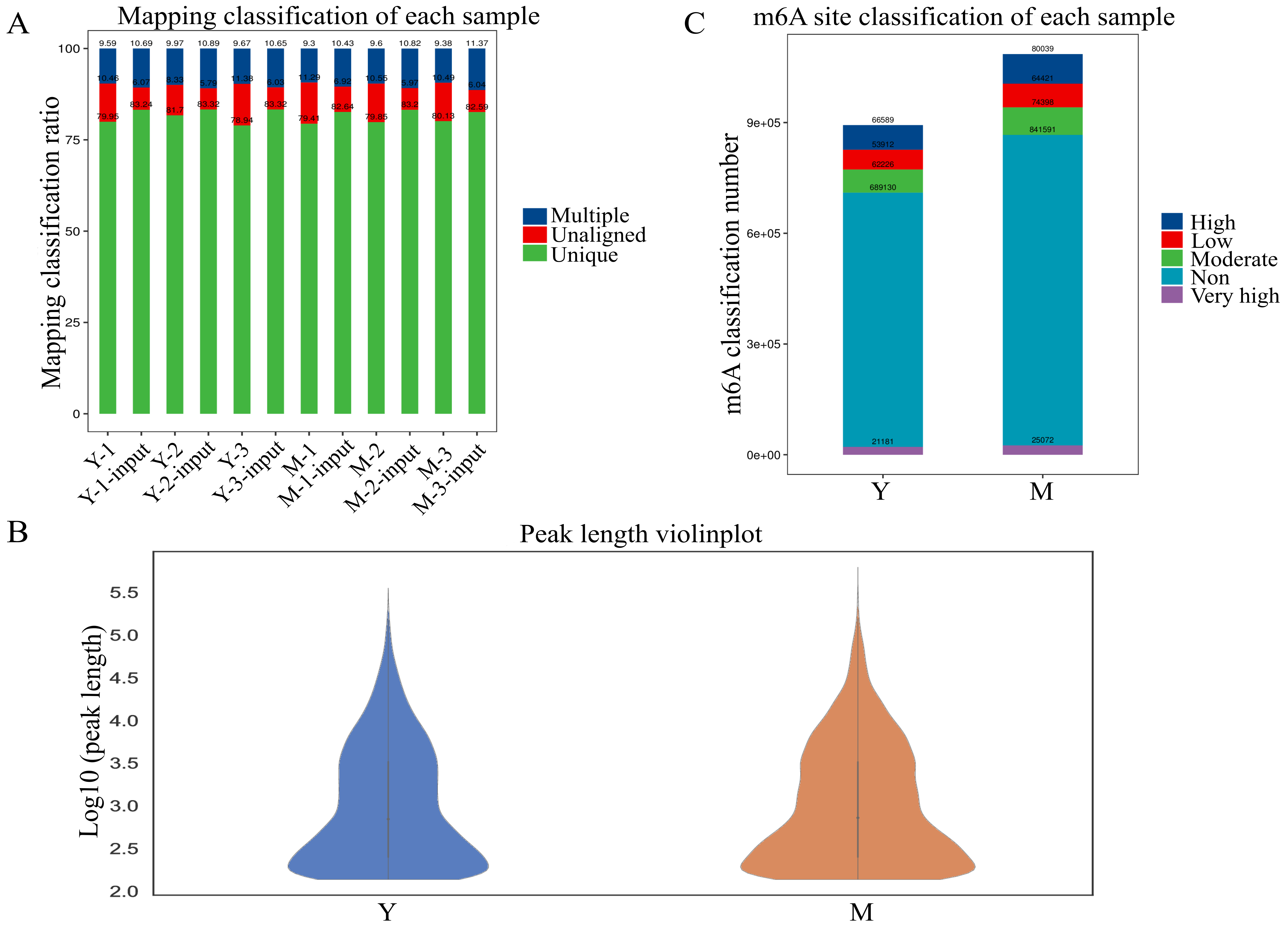

Supplementary figure S1: Statistical analysis of sequencing data. (A) Mapping classification of each sample; Y: Before sexual maturity, M: After sexual maturity, input: Common RNA-seq library. Input, as the background, is used to correct the expression level of RNA in different samples, so as to make the peaks more accurate. (B) Peak length violin plot. (C) m6A site classification of each sample.
